# Odontogenic Sinusitis: From Diagnosis to Treatment Possibilities—A Narrative Review of Recent Data

**DOI:** 10.3390/diagnostics12071600

**Published:** 2022-06-30

**Authors:** Cristian Martu, Maria-Alexandra Martu, George-Alexandru Maftei, Diana Antonela Diaconu-Popa, Luminita Radulescu

**Affiliations:** 1 ENT Clinic Department, Grigore T. Popa University of Medicine and Pharmacy Iasi, Universitatii Street 16, 700115 Iasi, Romania; cristimartu@gmail.com (C.M.); lmradulescu@yahoo.com (L.R.); 2 Department of Periodontology, Grigore T. Popa University of Medicine and Pharmacy Iasi, Universitatii Street 16, 700115 Iasi, Romania; 3 Department of Dento-Alveolar Surgery and Oral Pathology, Grigore T. Popa University of Medicine and Pharmacy Iasi, Universitatii Street 16, 700115 Iasi, Romania; 4 Department of Oral Implantology, Removable Dentures and Technology, Grigore T. Popa University of Medicine and Pharmacy Iasi, Universitatii Street 16, 700115 Iasi, Romania; antonela.diaconu@umfiasi.ro

**Keywords:** rhinogenic sinusitis, odontogenic sinusitis, diagnosis, bacteria, dental treatment, antibiotic treatment, endoscopic surgery, Schneiderian membrane

## Abstract

The maxillary sinus is a structure at the border of specialties: otorhinolaryngology and maxillofacial surgery. Due to this fact, regarding etiology, it can be affected by both the rhinogenic and odontogenic path and can impose diagnostic difficulties. The etiopathogenic mechanisms that can affect the Schneiderian membrane are mainly inflammatory, iatrogenic, traumatic, and tumorous in nature. From a microbiological point of view, the bacteriology is polymorphic, including both aerobic and anaerobic species in acute OS, the predominating species in acute OS being aerobic, and in chronic anaerobic germs. The role of fungi in the determination of this pathology and in the production of the biofilm that leads to resistance to antibiotic treatment is also discussed. The present paper aims to present the etiopathogenesis, bacteriology, clinical manifestations, as well as treatment of odontogenic sinusitis (OS) from an updated perspective through reviewing the literature. If unilateral maxillary sinusitis is usually due to odontogenic causes, this does not clinically exclude the possibility of strictly rhinogenic causes in the occurrence of sinusitis. This underlines the important role of complex oral and rhinological clinical examination as well as the role of preclinical examinations in specifying the certainty diagnosis. Simple radiography, orthopantomography, CT, and CBCT are compared in terms of diagnostic accuracy. The treatment of OS is complex, involving medication, dental, and surgical measures. The value of endoscopic surgery is emphasized, comparing its advantages over the classic Caldwell-Luc technique.

## 1. Introduction

Acute and chronic sinusitis are public health problems both because of their prevalence—chronic sinusitis has a prevalence rate of 10.4% in Europe [1] and 14% in the USA [2]—and because of the costs involved—about 6 billion dollars plus 2 billion dollars for treatments without a prescription annually in the USA [3]. Only approximate estimates can be made regarding the prevalence of acute sinusitis because most patients do not seek a specialist’s consultation.

Supposing that an upper respiratory tract infection is complicated with bacterial rhinosinusal infection implies the existence of approximately 20 million cases of acute bacterial rhinosinusitis annually, in just the USA [4].

In the majority of cases, maxillary sinusitis has a rhinogenic cause, but due to the relation of the maxillary sinus with the alveolar bone and projection of the roots of the canines, premolars, and (mainly) molars, it can also have a dental cause.

Sinusitis is the inflammation of the nasal sinus mucosa characterized by the presence of two or more symptoms, one of which must be [2]:

- nasal obstruction;
- anterior or posterior rhinorrhea ±;
- pain or facial pressure;
- hipo or anosmia ±.

Sinusitis can be classified according to different criteria:

*A.*  *Etiology:*
  - viral sinusitis;
  - microbial sinusitis;
  - fungal sinusitis.
*B.*  *Time—EPOS classification* [5]*:*
  - acute sinusitis—with a duration of symptomatology less than 12 weeks;
  - recurrent sinusitis—in which there are two or more episodes of acute sinusitis per year with complete resolution between episodes;
  - chronic sinusitis—with a symptomatology that lasts more than 12 weeks per year without complete resolution of the symptoms.
*C.*  *“The Clinical Practice Guideline” by Rosenfeld and Andes* [6] *for adult sinusitis:*
  - acute sinusitis—with a duration of symptomatology less than 2 weeks;
  - subacute sinusitis—with the duration of symptomatology between 4–12 weeks;
  - recurrent acute sinusitis—with four or more episodes of acute sinusitis per year without persistent symptomatology between episodes;
  - chronic sinusitis—with a duration of symptomatology longer than 12 weeks.
*D.*  *Trigger of sinusitis* [6]*:*
  - rhinosinusal—when the starting point is rhinogenic.
  - odontogenic sinusitis (OS)—when the starting point is odontogenic.

OS appears due to inflammation of the mucosa of the maxillary sinus characterized by two or more symptoms, one of which must be nasal obstruction or nasal discharge associated with pain or facial pressure and/or reduction or loss of smell for at least 12 weeks as a result of Schneiderian membrane perforation through dentoalveolar pathology [2].

Although OS was first described about 100 years ago, it remains an underappreciated and underdiagnosed condition [7]. Data from the literature related to its frequency compared to that of rhinogenic causes are discordant [8,9,10].

## 2. Epidemiology of Odontogenic Sinusitis

OS accounts for 10–14% of the total maxillary sinusitis, with reports of up to 40% [5,11,12,13,14]. Out of the total number of unilateral maxillary sinusitis, OS accounts for 75% [11].

OS is usually found in patients between 40 and 60 years old and has a slightly higher frequency in women: 57.7% compared to men 42.82%, with a 1:1.33 ratio in some studies [15,16,17,18]. Another study on 1077 patients with OS reported a female to male ratio of 1:1.4 [19].

## 3. Etiology

The particularity of an odontogenic maxillary sinusitis diagnosis is defined by two anatomical elements. The first is represented by the dental units, which are the triggering factor, and the second is the maxillary sinus. Both the teeth and the maxillary sinus are dynamic elements that change their characteristics throughout life and implicitly change the relationships between them.

The maxillary sinus appears as a cavity in the maxillary bone during intrauterine life and reaches a volume of 15–20 mL between the ages 12–14 years old, once the eruption of the upper permanent teeth is complete [20].

Throughout life, the relationship between the dental-periodontal units and the maxillary sinuses are in a continuous dynamic position, determined by the physiological or pathological changes to which the facial massif is subjected [21]. Individual factors related to the anatomy of the sinus floor may be added to these factors: absent floor with dental roots in the sinus cavity, or with the apex covered only by the sinus mucoperiosteum [22,23]. Another situation commonly encountered in partially or wholly edentulous persons is the pneumatisation of the sinus cavity, which can progress inferiorly, forming a recession towards the alveolar bone, the result being the existence of only a thin layer of alveolar bone between the sinus and the oral cavity [20].

The causes for the production of OS may be due to [12,13,16,24,25,26,27]:

- infectious causes—dental and periodontal pathology: dental caries, endodontic infection caused by deep carious processes that develops with pulp and periapical complications and sometimes through complex endoparodontal lesions with an infrabony periodontal pocket as a starting point;
- iatrogenic causes—the most common cause of OS (55.97%): incorrectly performed sinus lift procedures, dental implants with dimensions and insertion axis not adapted to the individual clinical features, foreign bodies (perforations during endodontic treatments, overfilling of root canals beyond the apex with filling materials such as zinc-oxid eugenol or gutta percha), dental extractions with or without pushing a fragment of the root into the sinus cavity, orthognathic surgery, labio-palatine cleft surgery, Le Fort osteotomies;
- odontogenic cysts with sinus involvement;
- traumatic injures of the maxillary bone;
- tumoral—in the case of neoplasms.

The information is described in brief in Table 1.

In a study conducted on 674 patients diagnosed with OS, iatrogenic sinusitis was reported in 65.3% of cases, apical periodontal pathology in 25.1% of cases, and marginal periodontitis in 8.3% of cases [27].

From the anatomical point of view, the roots of the second molar are closest to the sinus floor, with an average distance of 1.97 mm, followed by the roots of the first molar, then the third molar, the second premolar, and the first molar, located at an average distance of 7.5 mm [23]. A study points out that the teeth most frequently associated with the development of OS are the first molar in 35.6% of cases, the second molar in 17.4%, and the second premolar in 14.4% of cases [28]. This is most likely due to the fact that the first molar is more frequently affected by periodontal and endodontic pathology.

Another study, a meta-analysis on the aetiology of OS indicates the molar region as a trigger of the disease in 47.68% of cases [15]. The authors identify the first molar to be the most commonly involved, with an incidence of 22.51%; followed by the third molar with an incidence of 17.21%; and finally the second molar, with an incidence of 3.97%. The premolar region causes OS in only 5.96% of cases, with the second premolar (1.98%) most commonly involved. The canine was involved in only 0.66% of the total cases of OS. The right maxillary sinus was more frequently involved than the left one by 2%. Cases of bilateral OS are reported as being rare [15].

A study on 100 teeth regarding the relationship between the floor of the maxillary sinus and the underlying teeth revealed that the first molar is most commonly associated with the alteration of the floor of the maxillary sinus in 55% of cases, followed by the second molar (34%), second premolar (8%) and first premolar (3%) [29].

From an anatomical point of view, the mesiobuccal root of the second molar is closest to the floor of the sinus but the palatal root of the first molar is most commonly associated with perforation of the floor. One explanation for this would be that the time/age difference between the eruption of the two teeth has an influence on the severity of the alteration due to caries [28].

As previously stated, most authors recognize the iatrogenic mechanism as the most important cause of OS [15,23,24,29]. This can occur through various causes such as perforation of the sinus floor during dental extraction or during the mechanical treatment of endodontic treatment, dental ankylosis, as well as incorrect placement of dental implants, in orthognathic maxillary surgery, pre-prosthetic surgery, sinus lift, and sinus graft. In all these cases sinus infection can occur by colonization with bacteria from the oral flora [19].

Another possible cause is the oroantral fistula (osteomucosal communication between the oral cavity and the sinus or nasal cavity), this is usually iatrogenic and arises from extractions, ablation of intramaxillary cysts, surgery of labiopalatine clefts, or persistent periapical infection, which lead to the formation of a fistula through the necrosis of the sinus floor [13].

Surgical manoeuvres are suspected as a triggering factor for OS in 64% of cases [28], followed by infectious causes, with periapical pathology suspected in 18% of cases, periodontal disease in 10%, and other factors in 8%.

Dental implant placement was a consequence of OS in 37% of cases and 29.6% were due to dental extraction [30]. Even if the maxillary sinus cortex is perforated during implantation, as long as it does not exceed 3 mm the risk of mucosal perforation is small; therefore, it is unlikely that OS will occur [30]. A higher number of risky interventions in dental surgery in recent years has increased the incidence of iatrogenic causes of OS [15].

### Dental Infections as a Starting Point for OS

If we compare the incidence of dental infections, which is considerable, with the very low incidence of OS, we notice an obvious discrepancy. This is due to the fact that the floor of the maxillary sinus has a dense bone structure which is usually a barrier in the way of dental infection spreading. In contrast, the anterior wall of the maxillary sinus is thinner and is more easily penetrated, thus infections of the vestibular mucosa or fascial space infections are observed more frequently than OS [8]. However, odontogenic infections can drain into the sinus, especially in patients that have roots closer to the sinus [8].

There are many ways in which infection can reach the tooth apex. For example, deep complicated cavities that affect the dental pulp causing pulpitis and then periapical infections or through severe periodontal disease that spreads along the infrabony pocket and can even lead to secondary endodontic lesions [26,31]. If the infectious process is in contact with the Schneiderian membrane, it can lead to its inflammation, hypertrophy, and even rupture, causing the release of pro-inflammatory factors that cause edema, fibrosis, and cystic degeneration. In evolution, the infectious dental pathology can have an acute and invasive phase, during which the bacteria can spread directly in the surrounding tissues, stimulating a hypertrophic reaction in the Schneiderian membrane, and a chronic phase in which the lesion is characterized by an adaptive immune response [9]. These changes can occur even if there is an osseous wall that separates the dental apex from the sinus floor [31]. Moreover, germs from the dental pathological process, through microbial toxins, exacerbate inflammatory mediators and cause changes in ciliary activity. *Pseudomonas aeruginosa* plays a central role in the secretion of lipooligosaccharides, which causes ciliary immobility and failure. These factors, together with pro-inflammatory cytokines such as TNF-alpha, interleukin (IL)-8, and IL-13, cause cessation of ciliary activity [32], which explains the changes that occur in the maxillary sinus. Another theory is that the inflammatory effects induced by the odontogenic infection determine sensitization of the sinus mucosa and to the causes of infection of rhinogenic nature [16].

These histopathological features, in addition to the severe degree of inflammation observed in 73.9% of OS, may represent a stage of the disease that makes steroid and antibiotic treatments ineffective [33]. At the same time, it differentiates OS from maxillary sinusitis of rhinogenic origin from an etiopathogenic and therapeutic point of view.

Increased eosinophilia was observed in almost 40% (39.1%) of OS [33]. This is considered to be a risk factor both for developing OS and for its subsequent evolution. These patients considered at risk should benefit from antibiotherapy, dental treatment, and surgical treatment, preferably endoscopic [33], in order to restore healthy sinus mucosa. Both anatomic and functional aspects have to be addressed to rehabilitate the indispensable ciliary function, for proper functionality of the rhinosinusal mucosa.

## 4. Microbiology

The bacteriology of OS is different from that of non-odontogenic sinusitis [34]. The range of microbial species found in OS (acute or chronic) differs from that found in maxillary sinusitis of rhinogenic origin: *Streptococcus pneumoniae*, *Haemophilus influenzae* and *Moraxella catarrhalis*. These are predominant bacteria in acute rhinogenic maxillary sinusitis but are almost always absent in OS. The microbial flora from OS is polymorphic, in which anaerobic germs predominate [35,36].

The flora of acute OS is represented by aerobes such as *Hemolytic Streptococcus alpha*, microaerophilic streptococci, *Staphylococcus aureus*, and *Streptococcus pyogenes*, but also by anaerobes such as Gram negative bacilli, *Peptostreptococcus*, *Fusobacterium sporulatum*, and *Propionibacterium acnes* [36].

The flora of chronic OS is characterized by the predominant presence of anaerobes such as Gram-negative bacilli, *Peptostreptococcus*, and *Fusobacterium* spp., but aerobes can also be encountered, such as *Streptococcus c. alpha-hemolytic*, *Streptococcus c. microhemolytic, Staphylococcus aureus* [36]. Various studies regarding the bacterial species involved report the presence of more than 158 species and several fungi species [37,38,39].

The similarity between the germs of the oral cavity and those in OS is observed in many studies, which explains the prevalence of anaerobes in OS [11,40,41,42,43] and the large variation of the flora of the periapical infections involved in the aetiology of these infections. Intraradicular bacteria and fungi that may cause secondary periapical lesions are *Streptococcus, Propionibacteriu*, and *Candida* spp., in particular, *Candida albicans* [16,41,42,43,44]. In periradicular pathology it is difficult to identify the aetiology of the lesions because the bacteria can be extraradicular and diffuse to the periapical area [38,42,43,44]. This fact is confirmed by bacteria commonly found in abscesses or fistulas, which are consequences of secondary periapical lesions [44,45]. Another microbial agent that causes endodontic infections and can cause maxillary sinusitis of odontogenic origin is *Aspergillius* [46].

In this regard, the role of polymicrobial biofilm is intensely discussed in the literature, especially in cases of chronic OS resistant to treatment. Bacterial biofilms are dynamic polymicrobial communities of bacterial strains that replicate and have a constant metabolism and are incorporated into a matrix rich in exopolysaccharides, proteins, and nucleic acids [47,48]. Oral bacterial biofilms represent one of the most diverse and complex ecosystems, developed by successive colonization of over 700 bacterial species [49]. They adhere to the surface of teeth, gingiva, tongue, and other tissues of the oral cavity. After adherence to the tooth surface, bacteria progress from the supragingival to the subgingival area, transitioning from aerobic to anaerobic species, thus favouring the growth of anaerobic negative gram bacilli and limiting the growth of aerobic gram positive bacilli [49]. Microbes present at the supragingival and juxtagingival level are responsible for gingivitis and root surface caries, while subgingival species cause periodontal disease [50].

Biofilm formation occurs in three stages: (1) the adhesion stage, which begins with the formation of a film on the surface of the tooth followed by the initial bacterial colonization, (2) growth, and finally, (3) biofilm maturation and detachment [51].

Bacterial biofilms are involved in many chronic infections and are more difficult to eradicate due to their multi-layered structure. Microbes located in the deep layers are protected from the action of antibiotics or other antimicrobial agents [48]. Many authors have highlighted the presence of bacterial biofilm in chronic rhinosinusitis, considering that the resistance to treatment is precisely due to the particular structure of the biofilm. The presence of *S. aureus* within the biofilm is important because of its toxins, which cause immune system activation and an exacerbated anti-inflammatory response [32,50,51].

The extremely high diversity of microbial flora, most often in the penicillin-resistant category, makes it difficult to understand the complete picture of the ecology in OS and to specify an ideal antibiotic protocol [12,15,18].

The pathogenesis of OS is influenced by the relationship between the teeth roots and the floor of the maxillary sinus. The relationship between the maxillary sinus and the endodontic space regarding the penetration of infection from the pulp into the sinus was studied by Selden in endo-antral syndrome [52]. This is characterized by:

- pulpal pathology in a tooth with sinusal involvement;
- radiotransparency in the periapical area in the teeth with pulp involvement;
- lamina dura interruption in the inferior sinusal margin of the affected teeth with sinusal involvement;
- a radio-opaque supraapical finding invading the sinusal space representing the sinus mucosal involvement and hypertrophy;
- variable degrees of radioopacity of the sinusal space.

It is important to note that infection can extend to the maxillary sinus, not only because of the topographic closeness between the roots and the sinusal floor, but also by means of circulation due to the common vascularization between the sinusal mucosa and periodontal tissue and/or through the fascial spaces [53,54,55].

The severe inflammation seen in 73.9% of OS cases represents the advanced stage of disease, causing antibiotic treatment failure. The thickening of the sinusal membrane, present in 69.6% of OS, is the factor that decreases the efficiency of the medical and dental treatment [54].

## 5. Clinical Manifestations

As previously mentioned, odontogenic sinusitis develops through the violation of the Schneiderian membrane. In order to be able to make the differential diagnosis, a complex clinical examination is required, starting with taking a careful history (the onset can be difficult for the patient to place) followed by an inventory of symptoms and a general clinical examination.

Nasal obstruction syndrome is usually the first symptom, accompanied by purulent rhinorrhoea, purulent secretions visible on the posterior wall of the pharynx, facial and dental pain, fatigue, hyposmia, and bad breath. This clinical table is often incomplete, as sometimes the condition evolves asymptomatically [55,56]. From the aforementioned symptoms, the most frequent is purulent rhinorrhea, which occurs in 66.7% of cases [57]. Dental pain is often absent and if present in the absence of other symptoms, it is not specific for OS [8].

Clinical examination is important and includes the examination of the oral cavity, including the buccal vestibule, which can be congestive, oedematous, and inflamed. Palpation of the anterior wall of the maxillary sinus may also be painful; percussion of the maxillary posterior teeth with possible sinus involvement may provide clues for localizing the lesion and of the causative dental unit. Examination of the oral cavity continues with evaluation of teeth, the coronary integrity, the appearance of dental pulp, the periodontal tissue, the dental roots condition (evaluate the possibility of fractures at this level), the existence of dental implants, possible interventions of sinus lifting, and the presence of oroantral fistulas [16].

During the paraclinical examinations, an anterior rhinoscopy will be performed, highlighting the changes in the mucosa and the presence of pus in the nostril or the middle meatus. The purulent secretion from OS is often yellowish-green and fetid. Nasal endoscopy provides additional details, given the possibility of examination by optical magnification and at different angles, details that are difficult to observe by direct rhinoscopy [12].

The radiological examination is of particular importance in the diagnosis of OS. The revealing lesions are periapical osteitis, periradicular osteitis and thickening of the maxillary sinus mucosa. This could indicate the diagnosis of apical periodontitis, periodontal disease, and odontogenic maxillary sinusitis [17,58]. Apical periodontitis represents the inflammation of the apical periodontal tissues, possibly associated with a radiolucent apical area.

Periodontal disease is detected when the alveolar bone lesion is greater than 2/3 of the length of the root or of the interradicular area in teeth with multiple roots [59,60]. A thickening of the maxillary sinusal mucosa greater than 3 mm represents a pathological situation, even 2 mm in cases with associated with other symptoms [61]. Mucosal thickness can increase up to 15 times in maxillary sinusitis [54].

Periapical radiography allows for the detection of dental caries and periapical lesions, evidenced by radiotransparency. Due to it being a 2D examination, its value is limited in the case of multiple roots [62]. Panoramic radiography offers an overview of the entire maxilla, with the disadvantage of being less sensitive in detecting periapical lesions [63]. The advantage is the low cost and the limited irradiation dose for both methods. The sensitivity of dental radiographs in detecting carious dental lesions is 60% and 85% in detecting periodontal disease [62]. From the perspective of a correct radiological diagnosis, the advantage of using computer tomography (CT) or cone-beam computer tomography (CBCT) in the diagnosis of periodontal disease is clear. Up to 60% of periapical pathology can be omitted when using periapical radiographs when compared to CT [64]. The possibility of examining the sinusal-dento-alveolar complex, both in axial and coronary incidence on CT images, offers more details and accuracy to the diagnosis.

Refinement of CBCT technique brings a number of advantages over classic CT: higher resolution, lower cost, 10% lower irradiation dose when compared to CT, and easier to tolerate for the patient due to shorter examination time and a more comfortable position. CT can create artifacts if hyperdense materials exist in the examination area and it is also less accurate in providing details in examining periodontal and endodontic lesions [65,66]. The benefits of CBCT are evident not only from the arguments presented but also from a statistical point of view: dental infections were not evident in 86% of the initial radiographs whereas they could be detected in 67% of CBCT examinations [67]. However, as of yet conventional radiography remains the primary radiological technique [68]. This opinion is supported by the American Endodontic Association [69], due to the accessibility, the cost, and the low irradiation dose (irradiation through CBCT being 10 times higher).

CBCT is elect in complex cases from the diagnosis and treatment point of view, especially when it is necessary to specify the thickness of the maxillary sinus floor and to precisely evaluate whether there was any sinusal disease before implantation [68].

The correct diagnosis depends not only on the radiographic method, but rather more on the skill of the evaluator. The most accurate method is the CT and CBCT exam. However, sensitivity and specificity vary between 47–89% and 64.3–94.4% respectively, depending on the evaluator, even with this method [65].

## 6. Treatment of OS

Because OS is considered to be a maxillary sinus disease, located at the intersection of several medical specialties for optimal treatment, a close collaboration is necessary between ENTs, oromaxillofacial specialists, and dental specialists. The exact assessment of dental lesions, if they are present, will be followed by the appreciation of their involvement in the maxillary sinus, should that be the case [70]. Due to different causes (associated diseases, immunodeficiency, particularly virulence of germs), serious complications can occur that may threaten the patient’s life [71,72,73,74]. The importance of a thorough evaluation to establish the correct diagnosis is emphasized by results showing that 20% of patients with OS are not diagnosed correctly and only 33% have been cured after initial treatment [75]. Moreover, OS does not respond to antibiotic treatment in 79% of cases, thus endoscopic surgery is required [76].

Considering the increasing tendency of resistance to conventional treatment of OS, the need for an interdisciplinary consensus of OS is underlined [75].

The therapeutic algorithm usually includes 2 stages:

- non-surgical treatment: antibiotic treatment for the infection and resolution of dental lesions;
- sinus surgical treatment.

Sometimes antibiotic treatment for the infection with a resolution of dental lesions are sufficient to solve sinusal problems but most often, the surgical stage is still necessary. The predictive factors that determine the failure of drug treatment and therefore require dental or sinusal surgical treatment are still not fully understood [41]. This is also shown in a study of 55 patients, revealing that 10% of the subjects were cured only with drug treatment, 10% only with dental treatment, 33% only with endoscopic surgery and 33% were cured with dental and endoscopic surgery [77].

Regarding sinus surgery, endoscopic technique became the first choice, given the low rate of complications and morbidity [35].

- *Antibiotic treatment*

Antibiotic treatment should be prescribed in accordance with the antibiogram. If no antibiogram is available, ampicillin or piperacillin combined with a β-lactamase inhibitor is recommended [23]. Another treatment possibility is a combination of levofloxacin and vancomycin [23,78].

An alternative is represented by tetracyclines or fluoroquinolones [79,80]. Moxifloxacin has been documented as a sufficiently active antibacterial agent against anaerobes and with broad activity against gram-positive and gram-negative aerobes [15].

The duration of antibiotic treatment in OS should be at least 14 days or at least 7 days after the resolution of symptoms [78]. Some authors recommend antibiotic treatment for 21–28 days [70].

B. *Dental Treatment*

Elimination of the source of the dental infection is necessary in order to prevent the persistence of symptomatology [10,76,81]. Depending on the clinical situation, it implies the extraction or root canal treatment/retreatment of an infected tooth or the extraction of a root from the sinus. Root canal treatment requires biomechanical instrumentation (respecting the anatomical and biological properties of the root) for the mechanical preparation of the affected root canals, effective disinfection, and filling of said root canals with an appropriate filling material [41]. Root-end surgery (or apicoectomy) may be performed in difficult-to-treat cases, or in the case of endodontic retreatment, where primary endodontic treatment was unsuccessful [41].

The existence of a non-infected dental fragment, without occurrence of a sinus mucoperiosteal perforation or with a size smaller than 3 mm, can be left in place. The patient will be monitored and treated with antibiotics and decongestants until the anatomical closure of the defect [81]. In contrast, if the perforation is larger than 3 mm, or in case of over infection, extraction is performed [76]. Additionally, a mucoperiosteal flap is elevated superior to the extraction region, the bone is drilled to form a window into the buccoalveolar recess, and the root is extracted. This technique is favoured because it widens the extraction area, especially in the posterior region of the maxilla (second and third molar), thus creating a wide oroantral communication. This can be closed primarily with a buccal flap, or in a secondary stage if the first closure technique fails. In this case, the Moczair sliding flap technique will be used [8]. If the patient has a dental implant with peri-implantitis that is the cause of OS, there is not a clear indication for implant removal; rather, peri-implantitis treatment should be ensued, plus endoscopic sinus surgery, and the patient monitored [38].

Oral fistulas can close spontaneously by blood clot formation if the defect is less than 5 mm [82]. In order to promote healing, it is recommended to cover the area with a protective absorbable material. In contrast, if the defect is greater than 5 mm, primary closure is required. The intervention should be performed in a sinus with healthy mucosa and infection control [8].

The problem of healing only by dental treatment raises various controversies. Many patients with reduced inflammatory phenomena can heal only by the resolution of the dental pathology but in cases with structural abnormalities such as oroantral fistulas, odontogenic or inflammatory cysts, foreign bodies that determine changes that characterize OS, sinusal surgery is required [83,84]. Some studies have shown that patients who underwent sinusal surgery first, followed by oral surgery have the same percentage of healing with those that first underwent dental surgery [9,10,35,41,70].

C. *Sinusal surgical treatment*

Sinusal surgical treatment is reserved for foreign intrinsic bodies, whether they are represented by included teeth or by tooth roots displaced in the maxillary sinus that have caused maxillary sinusitis [85]. There are multiple procedures by which the maxillary sinus can be approached, but these derive from the Caldwell-Luc intervention.

The operative times in the classical intervention described by the authors are [85]:

- incision in the gingivolabial groove;
- deperiostation of the canine fossa;
- milling at this level to elevate and remove the nasal mucosa;
- verification of the projection area of the teeth at the sinusal level;
- the breach in the medial wall of the maxillary sinus at the level of the inferior meatus for drainage.

In 1988, Defreitas and Lucete [86] reported that of 670 Caldwell-Luc interventions performed, there were complications in 522 of them, such as facial swelling in 89% of patients, discomfort in the genian region in 33%, temperature higher than 101 °F in 12%, and significant bleeding in 3%. Late complications were facial asymmetry in 0.7% of patients, facial paresthesias in 9%, oroantral fistula in 1%, dehiscence of gingival wound in 1%, dacryocystitis in 2%, dental devitalization in 0.4%, recurrent sinusitis in 12%, and recurrent polyposis in 5%.

Although it has many disadvantages (the need for hospitalization days, general anesthesia, high costs, and as presented above, the possibility of numerous complications), the Caldwell-Luc technique is still extensively used as such or in a modified version [87,88]. It should also be emphasized that the sinusal mucosa loses its clearance function and thus sinusal drainage can no longer be performed physiologically [87,88]. The advantages of the Caldwell-Luc technique are that they offer a wide view, which allows for the extraction of sinus foreign bodies, cysts, and tumours. Other indications are related to facial trauma, necrosis of the jaw, and the existence of a fungus ball [88,89].

In case infection or tumour formation has spread to the neighbouring sinuses or to the pterygomaxillary fossa, the Caldwell-Luc technique is used as the technique of choice. It is worth noting that in recent years, even in difficult cases of large tumours and fungus balls, experienced endoscopists still prefer the endoscopic technique [90].

Endoscopic sinus surgery aims to eliminate infection through excision and removal of materials, teeth, and cysts, but also to restore proper drainage and sinusal ventilation. Through maxillary antrostomy, the ostium of the maxillary sinus can be enlarged [35].

When compared to the Caldwell-Luc technique, endoscopic sinus surgery has clear advantages, such as minimal incisions, no scar in the oral cavity, reduced hospitalization time, minimal invasiveness, offering the possibility to treat the odontogenic source and concomitantly solve sinusal problems while maintaining the functionality of the maxillary sinus, and a much lower rate of complications [91]. Felisati [92] treated 220 patients endoscopically with a 99% success rate, but the author simultaneously treated the odontogenic source and the sinus infection. Dundar [93] and Safadi [94] used the endoscope to extract implants from the maxillary sinus.

Nasal endoscopy is performed with general and local anaesthesia with epinephrine in the intervention area in order to minimize bleeding. The rigid 0° endoscope penetrates 4 mm, usually in the middle meatus and, after the uncinectomy, the ostium of the maxillary sinus is identified, and it is widened posteroinferiorly so that the sinus can be observed [14]. Angular endoscopes of 45° and 70° can be used to visualize the lower maxillary recess. In type II sinusotomy, the maxillary sinus is opened no more than 2 cm in posteroinferior diameter. In type III sinusotomy, the antrostomy is extended at the level of the posterior wall, anterior to the lacrimal sac and inferior to the base of the inferior cornice [95,96]. Depending on the situation, if there are lesions at the level of the other ethmoidal sinus, sphenoid or frontal sinuses, the endoscopic procedure may continue [96]. If there is an oroantral fistula, a buccal mucoperiosteal flap can be harvested from the posterolateral side of the maxilla in order to have access to the alveolar process [14]. In the case of an osteitic process, the area is drilled by removing the affected bone. The sinus is inspected by endoscope, so that areas with osteitis are removed, the sinusal mucosa is washed with rifampicin, and if the oral communication is very small (0.5–0.8 mm), the communication is closed with a simple mucoperiosteal flap [97].

If the communication is bigger, a buccal pedicle flap harvested from the posterolateral region of the maxilla is transferred to the fistula. It is fixed in the correct position by 2–3 points of suture in the maxillary side wall, taking care that there is no tension [93]. This flap is covered with a flap of vestibular mucosa, making incisions in the periosteum so that there is no tension. The patient will remain in the hospital 24 h postoperatively, the nasal tamponade is removed at 48 h, and an antibiotic treatment (Augmentin 2 g/day or Levofloxacin 500 mg/day) will be administered for 14 days postoperatively [14]. It should be noted that there are differences between the oroantral communication that represents an osteomucosal pathological union between the oral cavity and the maxillary sinus as a result of medical procedures or pathological processes and the oroantral fistula which represents the chronic form of oroantral communication [98,99].

The difference between oroantral communication and oroantral fistula is represented by the presence of the squamous epithelium that comes from the oral mucosa and the pseudostratified ciliary epithelium that comes from the sinusal mucosa, elements that characterize oroantral fistulas [100].

Most small oroantral communications with a diameter between 1–2 mm without epithelialization close spontaneously [100]. Sroantral fistulas ≥ 5 mm in diameter persisting for more than 3 weeks require surgery [99]. Factors that prevent spontaneous closure include epithelialization of the fistula pathway, osteitis of the communication margins in dental abscesses, cysts, foreign bodies, and tumours, which causes the formation of a chronic fistula. Persistence of sinusal infection and oral communication for more than 3 weeks recommends closure of the fistula even if it is less than 5 mm [100].

For the closure of an oroantral communication, different techniques are used, chosen according to localization of the communication, local anatomical conditions, evolution time of the oroantral communication, presence of inflammation in the paranasal sinuses, and general condition of the patient [96]. Among the techniques used, we mention sliding vestibular flaps, palatal flaps for rotation and transposition, lingual flaps, and temporal muscle flaps [101]. Of these, the flap of Bichat’s fat pad is recommended for the closure of oroantral fistulas and other oral defects [101,102].

Bichat’s fat pad consists of a central lobed mass formed by three lobes: anterior, intermediate and posterior. The posterior lobe has four digital buccal extensions, pterygoidian, pterygopalatine and temporal, which extend into the respective areas [102]. Buccal extension is the used most frequently for closure of oroantral communications due to its anatomical features [103]. Histologically, Bichat’s fat pad is different from adipose and subcutaneous tissue and is composed of the same type of fatty tissue as orbital fat [104]. Its volume is constantly independent of the distribution of body fat of the individual [105]. It has a volume of 9.6–10 mL and a weight of 9.3, with a width of 6 mm being able to cover defects of small and medium size of about 4 cm [105]. Bichat’s fat pad has a rich blood supply, with arteries coming from the maxillary artery, superficial temporal artery, and branches from the facial artery [104].

The advantages of using this flap are represented by easy access, rich vascularization, good mobility, complete epithelialization, low complications, and low risk of infection [103]. The disadvantages are its size, which can cover defects of only 5.0 × 4.0 cm as closing a larger surface would cause tension in the flap, which would be deprived of blood and lead to poor vascularization and dehiscence of the wound [103,106]. To avoid complications, preoperative MRI evaluation is recommended to determine the total volume of Bichat’s fat pad and to avoid complications [96].

Complications of this flap include partial necrosis, infections, aesthetic changes of the cheek, lesions of the facial nerve, hematoma, hemorrhage, paresthesias of the buccal nerve, and recurrence of FOA and are reported in the literature at a rate of 3.1–6.9% [104,105,106]. To avoid them, a good technique is necessary; the sutures need to be made without tension and the flap must adequately cover the oroantral fistula [106].

Treatment of OS is complex and each patient will be treated individually according to the etiopathogenic factors after thorough assessment of the situation according to the clinical and radiological examination.

From the point of view of the impact on quality of life, chronic sinusitis is comparable to other severe chronic diseases such as heart disease, diabetes, and chronic lung disease [107]. Furthermore, chronic OS is associated with a lower quality of life when compared to chronic rhinogenic sinusitis. This is due to the fact that chronic sinonasal symptomatology in chronic OS has a higher impact on quality of life than in patients with chronic rhinogenic sinusitis [108].

## 7. Conclusions

Even though odontogenic sinusitis is a relatively frequent pathology, especially in certain age groups, it is still underdiagnosed due to its non-specific symptomatology. Furthermore, it can be frequently confused with rhinogenic sinusitis, thus escaping appropriate treatment. For proper diagnosis and therapeutic management, it requires the collaboration of an ENT specialist, a dentoalveolar or maxillofacial specialist, and a dental specialist. Treatment can be complex, usually involving two stages: a non-surgical and a surgical stage; however, it is imperative that the dental pathological process is resolved, otherwise an efficient, complete treatment is not possible.

## Figures and Tables

**Table 1:** Causes of perforation of the Schneiderian membrane that may cause odontogenic sinusitis.

Causes	
**Infectious**	Dental pathology—endodontic infection
	Periodontal pathology—infrabony periodontal pocket
	Complex endoperiodontal lesions
**Iatrogenic**	Incorrectly performed sinus lift procedures
	Incorrectly placed dental implants
	Peri-implantitis
	Faulty endodontic treatment
	Dental extractions
	Surgical procedures: orthognathic surgery, labio-palatine cleft surgery, Le Fort osteotomies
**Odontogenic cysts**	with sinus involvement
**Traumatic injures**	of the maxillary bone
**Tumors**	


## Data Availability

Not applicable.
